# Soil microbial community differences drive variation in *Pinus sylvestris* physiology, productivity, and responses to elevated CO_2_

**DOI:** 10.1186/s40793-025-00828-w

**Published:** 2025-12-04

**Authors:** Mark A. Anthony, Nora Röckel, Alexandra Traistaru, Aswin Krishna, Henning Meesenburg, Markus Wagner, Frank Jacob, Arthur Gessler, Peter Waldner, Marcus Schaub, Marco Ferretti, Andreas Schmitz, Pim van den Bulk, Arjan Hensen, Stefan F. Hupperts, Lalasia Bialic-Murphy, Colin Averill

**Affiliations:** 1 Center for Microbiology and Environmental Systems Science, University of Vienna, Vienna, Austria; 2 Department of Environmental Systems Sciences, ETH Zürich, Zürich, Switzerland; 3 Northwest German Forest Research Institute, Göttingen, Germany; 4 Sachsenforst State Forest, Pirna OT Graupa, Germany; 5 Swiss Federal Institute for Forest, Snow, and Landscape Research (WSL), Birmensdorf, Switzerland; 6 North Rhine Westphalia Office for Nature, Environment and Climate, Recklinghausen, Germany; 7 Thünen Institute of Forest Ecosystems, 16625 Eberswalde, Germany; 8 The Netherlands Organization for Applied Scientific Research at Petten, Petten, The Netherlands; 9 Department of Forest Ecology and Management, Swedish University of Agricultural Sciences (SLU), Umeå, Sweden; 10 Funga Public Benefit Corporation, Austin, TX USA

## Abstract

**Background:**

Soil microbial communities can affect plant nutrient uptake, productivity, and may even confer resistance to global change. Elevated atmospheric CO_2_ is widely expected to stimulate plant productivity; however, this will depend on the availability of growth limiting nutrients such as nitrogen. Soil microbial communities are the main mediators of soil nitrogen cycling and should therefore play a key role in influencing plant responses to elevated CO_2_.

**Results:**

To test this, we conducted a controlled, growth chamber experiment with *Pinus sylvestris* to evaluate how soil microbiome variation influences plant physiology, productivity, and responses to elevated CO₂ (eCO₂; 800 ppm versus 400 ppm in the ambient treatment). Field soils were collected from six forests with varying tree growth rates and were used as an inoculant source, either sterilized or living, into a common growth medium seeded with *P. sylvestris*. After seven months of growth, we measured plant carbon assimilation, photosynthetic nitrogen use efficiency, above- and belowground productivity, and we measured soil microbial biodiversity using DNA metabarcoding. Our findings demonstrate that seedling productivity was stimulated under eCO_2_ conditions and that this was supported by improved plant photosynthetic nitrogen use efficiency, but only in the presence of living versus sterilized soil inoculant. The magnitude of this response was also dependent on the forest soil microbial inoculant source and was linked to a 70% increase in bacterial species richness, increased relative abundances of bacteria known to have positive effects on plant growth (e.g.,* Lactobacillus*,* Bacillus*,* Flavobacterium*), and with a concomitant shift in saprotrophic fungal community composition and root growth. Variation in inorganic nitrogen cycling which favored the accumulation of nitrate under eCO_2_ was also correlated with a twofold reduction in photosynthetic nitrogen use efficiency, suggesting a decoupling of nitrogen availability and assimilation efficiency with distinct implications for plant growth responses to elevated CO_2_.

**Conclusions:**

Our results show that soil microbial community variation directly affects *P. sylvestris* physiology, productivity, and responses to eCO_2_, and may enhance plant growth through improved nitrogen use efficiency. Surprisingly, growth with different microbial communities even more strongly impacted plant productivity than a doubling of atmospheric CO_2_ concentrations. The soil microbiome therefore plays a key role in supporting plant nutrition and growth under ambient and eCO_2_ conditions, and in turn, may confer increased forest resistance to climate change.

**Supplementary Information:**

The online version contains supplementary material available at 10.1186/s40793-025-00828-w.

## Introduction

Cross-kingdom interactions between plants and soil microbes may affect future forest productivity and adaptations to climate change. Plants worldwide will need to adopt new physiological strategies to sustain growth in a changing world [1]. Some global changes, such as elevated CO_2_ (eCO_2_), induce progressive nutrient limitations [2] which limit plant growth [3]. Plants can either “do more with less” by increasing their nutrient use efficiency or they can acquire supplementary nutrients [4]. Both strategies may be achieved through changes in their root systems or via interactions with mutualistic mycorrhizal fungi [5] and rhizosphere bacteria [6]. However, these changes may be constrained by physiological trade-offs. Increasing nutrient use efficiency is directly tied to water availability because nutrient uptake requires regulation of water loss via stomatal conductance [7], and if plants invest more into roots, this requires auxiliary allocation of energy from above to belowground, which may be limited by carbon assimilation [8]. How plants optimize their physiology in relation to the soil microbiome remains an open question, even though it may be important for sustaining forest productivity in a changing world.

Atmospheric CO_2_ levels have increased by ~ 50% since the industrial revolution [9–11], and they are projected to double again by 2100 [12], with significant implications for forest productivity. Elevated CO_2_ can boost plant growth [13, 14], but this requires sufficient nutrients, especially nitrogen (N) in temperate and boreal forests. In these systems, CO_2_ enrichment experiments demonstrate that N often becomes progressively less available to plants over time [2, 15]. To cope with declining N availability plants can enhance their photosynthetic N use efficiency (PNUE), defined as the amount of carbon assimilated per unit leaf N. Under eCO_2_, PNUE often increases, enabling plants to partially compensate for reduced N supply [16]. This shift reflects an internal optimization of resource use rather than enhanced nutrient acquisition and may allow for sustained productivity under nutrient-limited conditions [1, 15]. Microbial interactions could play a key role in supporting such physiological adjustments [17]. For example, the uptake of N and its efficiency of incorporation into biomass in agricultural plants is positively correlated with soil bacterial taxonomic richness [18] and stimulated by direct plant-growth promoting bacterial inoculations [19]. While these are both indicators of PNUE adjustments, the extent to which soil microbiomes support PNUE regulation and thereby contribute to plant growth under changing environmental conditions remains poorly understood.

To test this, we established a unique eCO_2_ experiment to isolate the effects of different forest soil microbiomes on plant productivity, plant physiology, and soil inorganic N cycling under ambient and eCO_2_ conditions. We focused on the Eurasian pine species, *Pinus sylvestris* L. (Scots pine), and we sourced soil communities from six distinct *P. sylvestris* forests along a steep gradient of tree growth rates in central Europe (Table 1). This sampling technique enabled us to include previously studied soil microbial communities from long-term forest monitoring plots, where we have identified links between the microbial community and tree growth rates [20, 21]. *P. sylvestris* is globally the most widespread pine species [18]. It also responds positively to eCO_2_, and earlier research has shown that this response can vary depending on its associations with particular ectomycorrhizal fungal species [22, 23], a key group of symbiotic fungi which inhabit plant roots and promote nutrient uptake [24]. How *P. sylvestris* responds to eCO_2_ in relation to differences in the soil microbiome more broadly versus individual fungal isolates in the lab has not been explored. We therefore developed a plant-growth chamber experiment to test whether microbial presence versus absence, microbial biodiversity, and microbial community composition were linked to underlying variation in seedling photosynthesis, water use efficiency, PNUE, plant productivity, and investment in above versus belowground biomass production.

We first hypothesized that (1) seedling productivity and responses to eCO_2_ would vary depending on the presence versus absence of soil microbial communities and with differences in the source of the soil microbiome inoculant, and (2) that these plant developmental metrics would be correlated with in situ tree growth rates from the locations where microbial inoculant was sourced. In other words, we hypothesized that microbiomes sourced from faster growing forests would stimulate seedling growth and responses to eCO_2_ more than microbiomes sourced from slower growing forests. We propose this hypothesis because previous research has demonstrated that inoculating soil can steer plant assembly towards the composition of vegetation from the soil inoculant source [25] and that soil inoculations can directly modify plant growth rates [26]. To our knowledge, there is no previous studies to test whether in situ tree growth rates from donor forests can predict the effects of inoculation on seedling growth, so our second hypothesis is more exploratory than the first.

## Materials and methods

### Site locations, soil sampling, and experimental design

We selected six ICP Forests (ICPF) Level II plots spanning a spectrum of tree growth rates with known dissimilarity in microbial community composition [20]. These sites were specifically selected because they harbor distinct ectomycorrhizal fungal communities (see Supplementary Fig. 1) and represent a 3.6 fold range in forest tree growth rates (Table 1). In August 2020, each plot was visited and 12 soil cores were collected along a 30 × 30 m grid using a tulip bulb corer (10 cm width × 10 cm depth). Cores were collected at least 1.5 m apart along the grid to sample a wide, representative area of each forest. Within each forest, all 12 cores were pooled, homogenized, and kept on ice in the field until being stored at 4 °C for 2–3 weeks prior to establishing the experiment. While planting into immediately sampled soil would be preferred, this cool storage period was unavoidable to sample every site and establish the full experiment. The full experiment included soil microbial inoculant sourced from six sites, a soil sterilization treatment (living versus sterilized microbial inoculant), a CO_2_ treatment (400 versus 800 ppm), and 20 replicates for each combination, resulting in a total of 480 sampling units.

**Table 1:** Site characteristics for the ICPF level II plots where soil was sourced for the mesocosm experiment

ICPF plot	Country^a^	Lat.	Long.	Altitude^b^	Tree Growth^c^	MAT^d^	MAP^e^	pH^f^	*N* depo.^g^
175	NL	51.19	5.31	≤ 50	0.53	10.02	743	4.16	2302
501	DE	52.34	11.1	≤ 50	0.41	9.83	833	3.69	3036
307	DE	52.54	7.51	≤ 50	0.33	9.04	749	4.06	2905
1405	DE	51.14	13.49	151–200	0.23	9.2	713	3.92	1470
901	DE	49.24	11.19	401–450	0.17	7.93	799	4.09	1948
9	CH	46.16	7.26	1051–1100	0.15	8.45	374	6.91	776

^a^Country abbreviations include the Netherlands (NL), Germany (DE), and Switzerland (CH)

^b^Meters above sea level

^c^Tree growth measured based on diameter at breast height growth increment (cm yr^−1^). A complete description of these details can be found elsewhere [20]

^d^Mean annual temperature (MAT) units (°C)

^e^Mean annual precipitation (MAP) units (mm yr^−1^)

^f^Soil pH measured at a 0–10 cm depth

^g^N depo = Nitrogen deposition and units are in mg N m^2^ yr^−1^

### Mesocosm establishment and growth conditions

We used a common growth medium and microbial inoculant strategy to pinpoint the effects of microbiomes on *P. sylvestris* growth. The base growth medium was unfertilized and consisted of potting soil (GO ON^®^ Blumenerde; 180 mg N/L, 300 mg P_2_O_2_/L, 650 mg K_2_O/L, 150 mg Mg/L) and 2 mm grain playground sand in 3:1 (v: v) ratio. It was sterilized at 120 °C for 20 min three times. Sterilization was confirmed by plating 1 mL of soil extract (1 g in 10 mL of DI water) on MMN plates with 2.5 g glucose L^−1^. We detected no microbial growth after one month at 18 °C, confirming effective sterilization (Supplementary Fig. 2). We also autoclaved half of the field-collected soil using the same sterilization procedure. This allowed us to test for potential effects of site-level differences in soil physical and chemical versus microbial characteristics. Field soil (both autoclaved and fresh) was then combined with the base growth medium in a 1:6 ratio (v: v), keeping each site separate for both treatments to ensure each site received its own living and sterilized soil inoculant. We filled pots (1 L) with 885 mL of growth medium and sowed ca. 8 cold-stratified (1 month at 4 °C) seeds 2 mm below the surface (seeds sourced from Tree Seeds Online Ltd, Rockcliffe, England).

Pots were then randomly arranged in two growth chambers separated by conditions of ambient and elevated CO_2_ (400 vs. 800 ppm) to approximate current and projections for 2100 [12], respectively. Plants were randomly rearranged and transferred on a weekly basis from chamber-to-chamber to avoid any intra- and inter-chamber effects. When this exchange occurred, we also switched the CO_2_ conditions, so both chambers were used for the ambient and elevated CO_2_ treatments. Thus, each plant was grown in both chambers and in different locations of each chamber. Chamber conditions were identical (save for CO_2_ levels) and simulated a 12-hour day: night light cycle. At 7:00 CET, growth lights (250 µmol m^−2^ s^−2^; 90% red, 10% blue) incrementally turned on until reaching 100% at 8:00 CET. At 19:00 CET, light incrementally decreased to 0% by 20:00 CET. Temperatures were 15º at night and ramped up to 20 °C as the lights turned on during the day. Humidity was set to 70%. Chamber CO_2_ levels were continuously monitored to maintain 400 and 800 ppm in the ambient and eCO_2_ treatments, respectively. Mesocosms were watered ca. 2 times per week with 100 mL of DI water. Mesocosms were never fertilized.

After 1–2 weeks of growth, mesocosms were manually weeded to 1–3 individuals per pot. There was no effect of the treatments on the number of seedlings that germinated, nor the number of seedlings maintained in each pot (*P <* 0.05). We did not weed every pot to a single individual because we wanted to have multiple plants in case of mortality. To test whether the number of stems in the pot affected above and belowground productivity, we re-ran statistical models (see below) that included number of stems as an additional co-variable alongside inoculation source, CO_2_, and sterilization. There was no effect of the number of stems on root growth (*P* > 0.05). For aboveground productivity, there was a significant four-way interaction (number of seedlings x inoculation source x CO_2_ x sterilization; *P* = 0.047). For the living inoculant treatment, there was no effect of the number of stems (*P* > 0.05). In the autoclaved treatment, there was a main effect of the number of stems (*P* = 0.0002), and counter intuitively, productivity increased with the number of stems per pot (see Supplementary Fig. 3). Importantly, there were no interaction terms with main effects (site and CO_2_) and the number of stems did not differ across sites nor between CO_2_ treatments (*P* > 0.05). We can therefore conclude that treatment effects were not driven by differences in the number of stems in any treatments, and that there was no effect on root growth nor mesocosms with living soil inoculant. In total, plants were grown for seven months prior to destructive harvesting. This timeframe was selected because plants in the sterilized microbial inoculant treatment were suffering from chlorosis. After accounting for mortality, we measured growth on 456 plants. Productivity was measured as total biomass production divided by the number of growing days.

### Leaf-level morphological and physiological traits and whole plant growth

To examine the effect of the elevated CO_2_ and microbiome community composition treatments on plant functional traits, resource use efficiencies, and biomass accumulation, we measured a subset of leaf-level morphological and physiological traits one month before destructively harvesting. The full list of traits included net photosynthetic rate under chamber conditions (A_net_), transpiration rate (E), stomatal conductance (g_s_), leaf carbon and nitrogen content, and leaf mass area (g m^2^).

A_net_ (hereafter ‘assimilation’), E, and g_s_ were measured using a portable photosynthesis system (LI-COR 6800, Licor, Lincoln, Nebraska, USA). The measurements were performed in the environmental chamber with a clear-top chamber (width of 1 × 3 cm). The physiological measurements were taken at ambient chamber light conditions, which was 210–400 µmol m^−2^ s^−1^. We set the CO_2_ limit to 400 ppm for both CO_2_ treatments before taking the physiological measurements. To minimize within-plant variability, we measured from the 2nd to 4th whorl of needles and minimized overlapping. The needles did not fully cover the entire measuring area. Leaf area was calculated using ImageJ^®^ software (Image Processing and Analysis in Java). We measured the specific leaf area (SLA) on five dried needle samples per plant from the second and third whorl. These same needle samples were then used to measure leaf carbon and nitrogen concentration on a CHNS analyzer (Vario EL Cube CNS Elementar Analyzer, Germany), which was used to estimate leaf N per unit leaf area (Narea). This subset of leaf samples was also used to characterize plant water use efficiency (WUE = A_net_ / g_s_; µmol CO_2_ mmol H_2_O^−1^) and photosynthetic N use efficiency (PNUE = A_net_ / N_area_; µmol CO_2_ g N^−1^ s^−1^). Because these measurements are slow, we only collected physiological measurements on a random subset of the plants from the living soil microbial inoculant treatment, and after removing data with measurement errors (which were not specific to any treatment), we obtained quality-controlled, physiological data from 117 plants.

### Sampling the mesocosms

Mesocosms were destructively sampled to measure plant biomass accrual above- and belowground, to sample the diversity and composition of the established soil microbiomes, and to measure soil inorganic N availability and net N mineralization. If more than one seedling was still alive in the plot, we selected the largest seedling to represent the mesocosm. We selected the largest plant to have a common selection criterion and because these were also the plants on which we conducted the physiological analyses. We removed each seedling using tweezers and measured the above- and belowground fresh mass. Fresh mass was then air-dried and measured again after one week to determine tissue moisture content. We examined the root system of each plant by eye and under a microscope (40 x magnification), and while we did not quantitatively score EMF root colonization, we observed EMF extensively along plant root systems in the living soil inoculant treatment (extensive forking, thickened fine roots with fungal mantles, extraradical mycelium). Soil from the pots was then homogenized, and from a subset of the replicates, a subsample was frozen at -20 °C for molecular analyses (2 g), air-dried to determine soil moisture content (5 g), stored at room temperature for soil pH (10 g), and stored at 4 °C to quantify inorganic N availability (10 g) within 48 h of sampling.

### Soil chemical analyses

Soil pH was measured on soil slurries in a 1:10 (mass: volume) ratio of soil and DI water using a pH probe. Soil ammonium and nitrate (inorganic N) concentrations were quantified < 48 h following soil sampling (Time 1) and after a seven-day laboratory incubation at room temperature and soil moisture levels of 50% (Time 2) to quantify net N mineralization on half of the sample replicates excluding one missing sample (*n* = 239). Inorganic N was extracted from soil (10 g) using 2 M KCl (40 mL) and quantified using a vanadium (III) reduction for nitrate and a modified Berthelot reaction for ammonium [27]. Net N mineralization was calculated as the difference of the sum of ammonium and nitrate at Time 2 versus Time 1.

### Molecular analyses

Microbiomes were characterized using 16S and ITS DNA metabarcoding to study prokaryotes and fungi, respectively. This analysis was only performed on a subset of the samples and after data QC to remove samples with low quality sequencing depth (see below), the analysis was conducted on 114 (16S) and 112 (ITS) samples. Genomic DNA was first extracted from frozen soil (250 mg) using the DNeasy PowerSoil Pro kit (Qiagen, Hilden, Germany) and was then used to amplify the variable regions 4 and 5 of the 16S rRNA gene using the primers 515F + 926R [28] to study prokaryotes and the entire ITS region using the primers ITS9mun + ITS4ng [29] to study fungi. These primers are not specific to fungi but rather amplify soil eukaryotes more generally. Each primer contained a 12 bp index sequence in the 5’ position. PCR reactions were performed in duplicate 25 µL reactions (13 µL of PCR grade water, 10 µL of Phusion Flash High-Fidelity PCR Master Mix, 1 µL 12.5 µM forward primer, 1 µL 12.5 µM reverse primer, and 1 µL of template DNA). 16 S amplicon thermocycler conditions were 94 °C for 3 min followed by 30 cycles of 94 °C for 45 s, 50 °C for 60 s, and 72 °C for 90 s, then 72 °C for 10 min, and finally a 4 °C hold. ITS amplicon thermocycler conditions were 95 °C for 15 min followed by 30 cycles of 95 °C for 30 s, 57 °C for 30 s, and 72 °C for 60 s, then 72 °C for 10 min, and finally a 4 °C hold.

The success and relative quantity of PCR product was assessed using agarose gel electrophoresis. We then pooled samples based on band intensity and removed remaining PCR reagents, short DNA and PCR products, and PCR primer dimers using AMPure beads for specific size selection. The ITS amplicons averaged ca. 750 bp whereas the 16 S amplicons averaged 300 bp. Pooled products were then quantified on a Qubit using the dsDNA BR Assay Kit (Invitrogen, Waltham, Massachusetts, USA) and sent for library preparation and sequencing at the Functional Genomics Center Zürich. 16 S libraries were sequenced using two Illumina MiSeq Runs with v3 chemistry (2 × 300 bp). ITS libraries were sequenced using two PacBio Sequel IIe SMRT Cell 8 M (15 h movie lengths).

### Bioinformatics

Raw sequences were first demultiplexed using Cutadapt [30] allowing for 0.10% mismatch, no insertions or deletions, and using the –pair-adapters function. 16S reads included forward and reverse reads whereas ITS sequences were single-end HiFi reads produced using the circular consensus sequencing mode.

Entire ITS regions were first extracted using ITSx (v1.1.3) [31] and then imported into QIIME2 for downstream processing [32]. There was no sequence-call filtering because the accuracy of HiFi reads provides a base-level resolution of 99.9% accuracy. We therefore dereplicated sequences and clustered *de novo* operational taxonomic units (OTUs) at 98% sequence similarity to account for variation in sequence conservation and capture ‘species’ identities, using the dereplicate-sequences and cluster-features-de-novo functions within the vsearch plugin [33] respectively. Singletons were later removed from the dataset in R. We assigned taxonomy against the UNITE (v8, 2021-10 release) database [34] using the naïve Bayes machine-learning classifier and the feature-classifier fit-classifier-naive-bayes function to train the classifier. We then assigned taxonomy using the classify-sklearn function and used the default confidence parameter of 0.7 [35]. We did not conduct an extensive set of statistics on the entire ITS-based dataset. The ITS primers we used target eukaryotes more broadly [29]), and after removing non-fungal sequences and singletons, approximately half of the replicates had lower sequencing depth than our rarefication cutoff (< 1000 sequences per sample) and were discarded.

Many remaining fungal OTUs were not assigned to a genus or species level ID, except for saprotrophic fungi, which were well represented in the remaining sequences (see Supplementary Fig. 4). Ectomycorrhizal fungi were always < 1% of the total sequences. This latter point is at odds with extensive visual ectomycorrhizal colonization observed on roots of seedlings with live but not sterilized inoculant (Supplementary Fig. 5). We attribute this to high amounts of amplifiable, non-fungal, eukaryotic DNA in the potting medium and perhaps most importantly, to sampling from the entire soil volume versus strictly rhizosphere soil or plant root systems to study ectomycorrhizal fungal communities. However, there were many detectable saprotrophic fungi in the ITS dataset, so we subset the data to focus on this guild and paid particular attention to the bacterial and saprotrophic fungal component of the soil microbiome.

Paired-end 16S sequences were analyzed using dada2 [36]. We first removed phiX and short reads (< 100 bp), truncated reads by removing primer sequences, and then discarded all reads with >2 expected errors and/or any ambiguous base calls. We then learned error rates, removed sequencing errors, merged forward and reverse reads, and removed chimeras using the learnErrors, dada, mergePairs, and removeBimeraDenovo (method = “consensus”) functions, respectively. We assigned taxonomy to amplicon sequence variants (ASVs) against the Silva database (nr_99_v138.1_wSpeciesTrain) using the assignTaxonomy function [37]. To account for unequal sequencing depth across samples, all data was rarified to 3,734 sequences per sample.

### Statistical analyses

All analyses were conducted in R and significance was set at *P* < 0.05. We used ANOVA to test the effects of inoculant sterilization (living vs. autoclaved), CO_2_ (400 vs. 800 ppm), different soil inoculant sources (i.e. the site from which inoculant was sourced), plus all two- and three-way interactions. The response variables included aboveground productivity, root growth, root-to-shoot ratio, and the physiological measurements (for physiology, we only tested the effect of different soil inoculant sources and CO_2_). We used the base *aov* and *Anova* function from the car package [38] to compute type ‘III’ sums of squares, and we estimated variation explained by computing partial eta squared (η^2^) as measures of an effect size. Normality of model residuals were always inspected. ANOVA was used for all univariate analyses (e.g., plant growth rate, microbial richness). Finally, we examined microbial and elevated CO_2_ impacts on plant development using Pearson correlations as cause and effect are difficult to disentangle in these types of studies. We calculated response ratios using the following formula:

$$ RR\;to\;the\;treatment = log10(Y_{{Trt}} /\user2{\bar{x}}(Y_{{Ctrl}} )) $$

where Y_Trt_ is the individual treatment replicate and x̄(Y_Ctrl_) are the control means. To test for the effect of microbial inoculant, Y_Trt_ was an individual replicate mesocosm with living inoculant, and x̄(Y_Ctrl_) was the mean value from all mesocosms with sterilized inoculant from the same site and CO_2_ treatment. To test the CO_2_ effect, each Y_Trt_ was an individual replicate mesocosm from elevated CO_2_ conditions and x̄(Y_Ctrl_) was the mean value from all mesocosms from ambient conditions of the same site and sterilization treatment.

To test the effects of inoculant sterilization, CO_2_, and inoculant source on microbiome composition, we used distance-based redundancy analysis and the *capscale* function in vegan [39]. This was only performed for the bacterial community due to limited coverage in the fungal dataset (see above). To correlate microbiome composition (bacterial and saprotrophic fungi) with mesocosm growth rates, root: shoot ratio, and physiology measurements, we used the principal coordinates analysis (PCoA) axes one and two as proxies for microbial composition, computed using the *pcoa* function in the ape package [40]. All permutation-based analyses were on rarified, relative abundance data using Bray-Curtis dissimilarity. We also computed species richness and Shannon diversity on the rarified datasets using the *specnumber* and *diversity* function in vegan, respectively. Lastly, species indicative of significant treatment effects were identified using indicator species analysis with the *multipatt* function in the indicspecies package [41]. Indicator species analysis was used to detect taxa associated to discrete levels of the significant predictor variables.

## Results

### Tree seedling growth is more responsive to microbiome variation than a doubling of atmospheric CO_2_

**Fig. 1:**
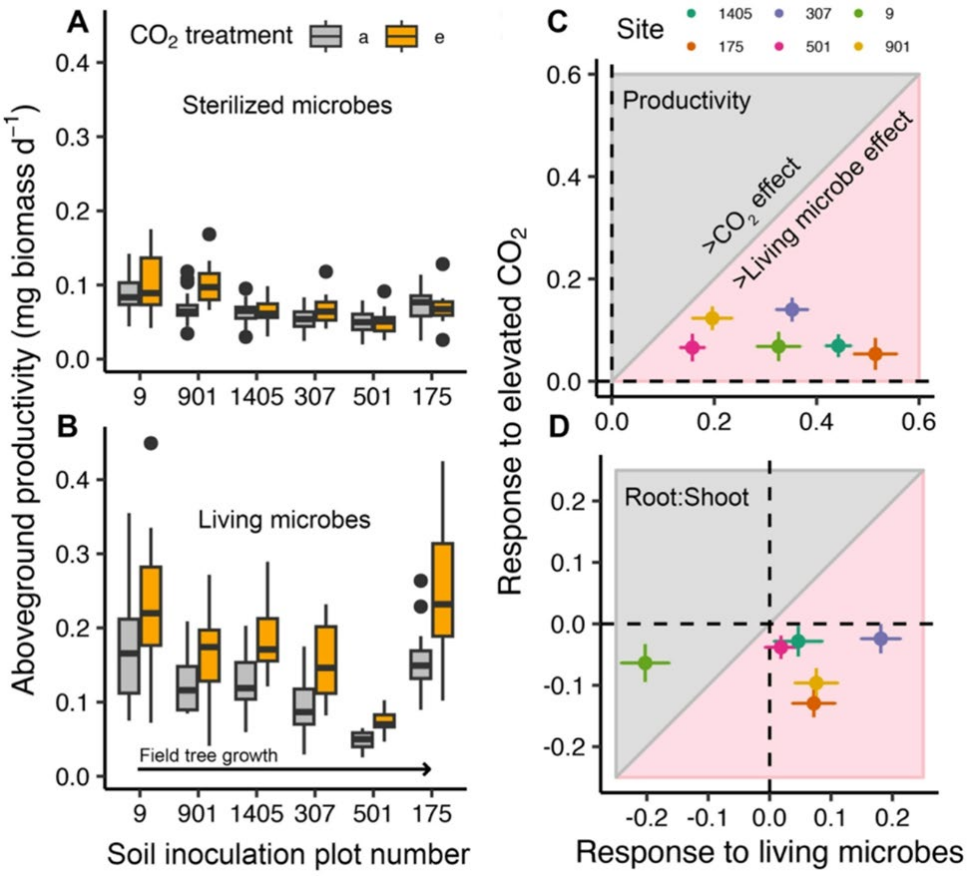
Tree productivity responses to elevated CO_2_ and soil inoculation sourced from six forests with variation in in situ tree growth rates. Aboveground plant productivity under ambient (a) and elevated (e) CO_2_ conditions in soils with sterilized **A** versus living soil inoculation referred to as “sterilized microbes” and “living microbes”, respectively (**B**). The x-axis represents the different sites, and it is ordered in relation to the average in situ tree growth rate of the sites from which the microbial inoculant was sourced (see Table 1 for a description of the different sites). Boxes represent the upper and lower interquartile ranges, whiskers represent the upper and lower ranges, and points represent outlying values. See Table S1 for a complete description of the statistical results. Plant productivity **C** and root: shoot ratio **D** responses to elevated CO_2_ in comparison to different sources of living microbial inoculation. Raw root: shoot ratio values are shown in Supplementary Fig. 6C, D. Points in the grey shaded areas represent larger effect sizes of elevated versus ambient CO_2_ in comparison to living versus sterilized soil inoculation while points in the pink shaded area represent larger effects of living versus sterilized soil inoculation in comparison to elevated versus ambient CO_2_. Points represent the mean response ratio for mesocosms inoculated from each individual site and error bars are the standard error

Aboveground productivity was 214% higher in soil with living versus sterilized soil inoculant (*P* < 0.0001; Supplementary Table 1; Fig. 1A, B) and 63% greater under elevated versus ambient atmospheric CO_2_ (*P* < 0.0001). A nearly identical pattern was observed belowground for root growth (Supplementary Fig. 6A, B) and for seedling root-to-shoot ratio (Supplementary Fig. 6C, D), which increased with living versus sterilized soil inoculant (*P* < 0.0001) and decreased under elevated versus ambient CO_2_ (*P* = 0.01). Aboveground productivity was most strongly affected by inoculation with living or sterilized soil (*η*^2^ = 0.45) followed by the source of soil inoculant (*η*^2^ = 0.32) and then eCO_2_ (*η*^2^ = 0.12). Growth with different soil inoculants therefore more strongly impacted tree development than a doubling of atmospheric CO_2_ concentrations (Fig. 1C, D).

Overall seedling productivity was homogenous across mesocosms with sterilized soil inoculant compared to mesocosms with different living soil inoculant sources (*F*-test: *F* = 0.13, *P* < 0.0001; Fig. 1A versus B). This demonstrates that there was a relatively negligible impact of soil physicochemical differences of the inoculated soil on plant growth; otherwise, there would be greater differences in the sterilized inoculant mesocosms across the source sites. In contrast, there was a strong impact of the biological variation of soil inoculant. This demonstrates that biological community differences, not differences in soil physical nor chemical characteristics of the inoculant, most likely caused variation in tree seedling productivity and growth responses to eCO_2_. Seedling productivity did not vary in relation to in situ field tree growth, as we hypothesized. There was even a negative correlation (Fig. 1B) between seedling productivity and in situ field tree growth rates until a breakpoint was observed at the fastest growing site (i.e., site 175).

### Tree physiology shifts with living soil inoculant source and photosynthetic nitrogen use efficiency can help explain why seedling productivity was stimulated by eCO_2_

**Fig. 2:**
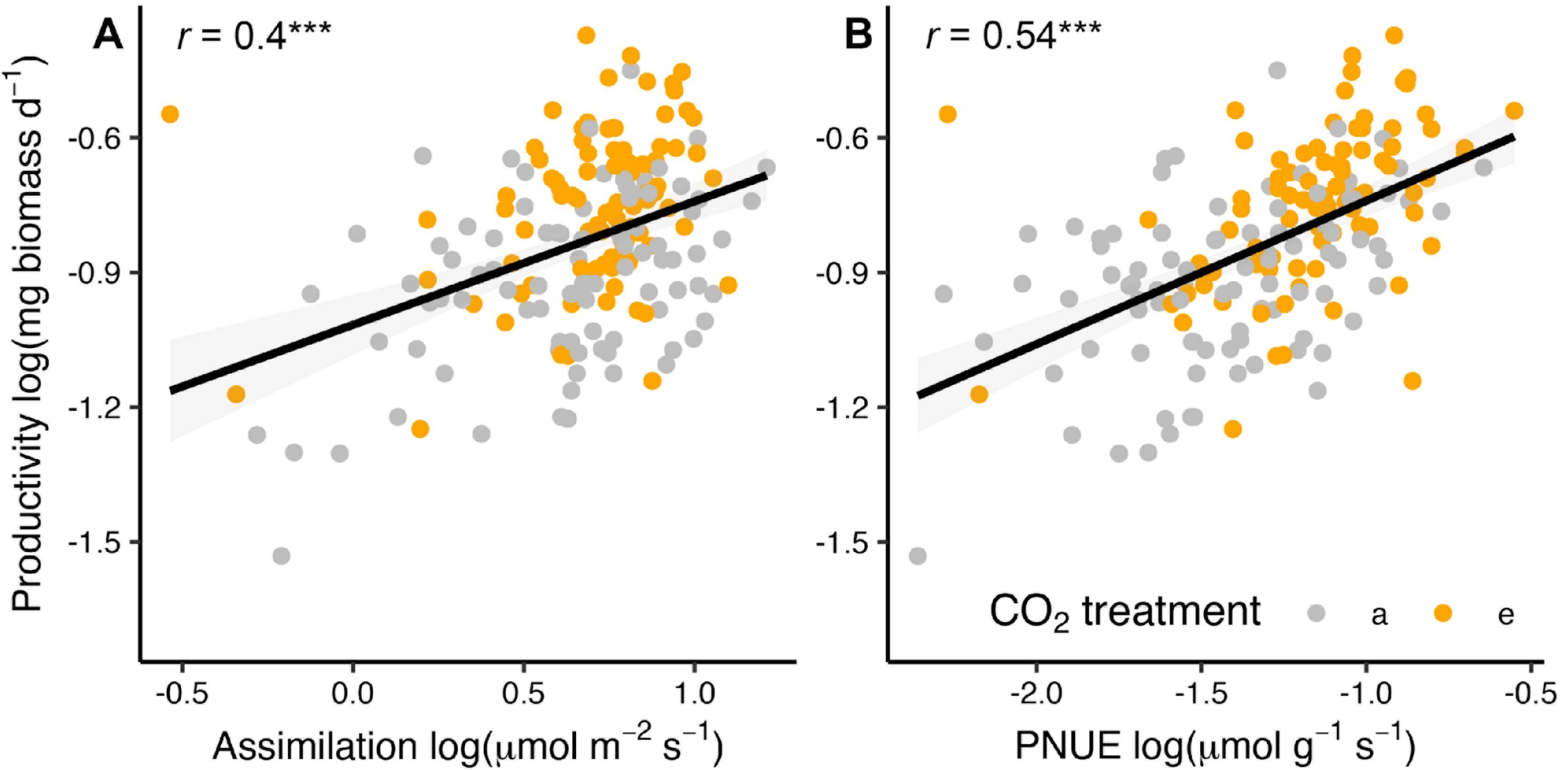
Links between tree productivity and physiological rates. Correlations between aboveground plant productivity and rates of carbon assimilation (**A**) and photosynthetic nitrogen use efficiency (PNUE; **B**) under ambient (a) and elevated (e) CO_2_. Results are only for mesocosms with living soil inoculant. Lines show linear correlations, shaded areas are 95% confidence intervals, *r* is the Pearson correlation, and asterisks indicate *p*-values (*** represents *P* < 0.0001)

Mirroring patterns in plant productivity, tree physiological rates varied with the source of living soil inoculant and were generally enhanced under eCO_2_. Carbon assimilation rate varied with the source of living soil inoculant (*P* < 0.0001; Supplementary Table 1) but not under eCO_2_ (*P* > 0.05). WUE was not impacted by the source of soil inoculant nor by eCO_2_ as individual factors, but there was a significant interaction between the two (*P* = 0.001). Conversely, PNUE varied with the source of soil inoculant (*P* = 0.02) and was 60% higher under elevated versus ambient CO_2_ (*P* = 0.01). Both assimilation (*r* = 0.4, *P* < 0.0001) and PNUE (*r* = 0.54, *P* < 0.001) were positively correlated with aboveground plant productivity (Fig. 2A, B), whereas WUE was weakly correlated (*r* = 0.2, *P* < 0.005), suggesting that higher assimilation rates increase plant biomass due to greater PNUE under elevated CO_2_ in our study system. Since all three plant attributes (i.e., productivity, assimilation, PNUE) varied with the source of living soil inoculant, we next explored whether any features of the soil microbial communities from the different soil inoculant sources could explain variation in these plant results.

### Bacterial communities vary across microbial inoculant sources, predict seedling growth and physiology, and are resistant to eCO_2_

**Fig. 3:**
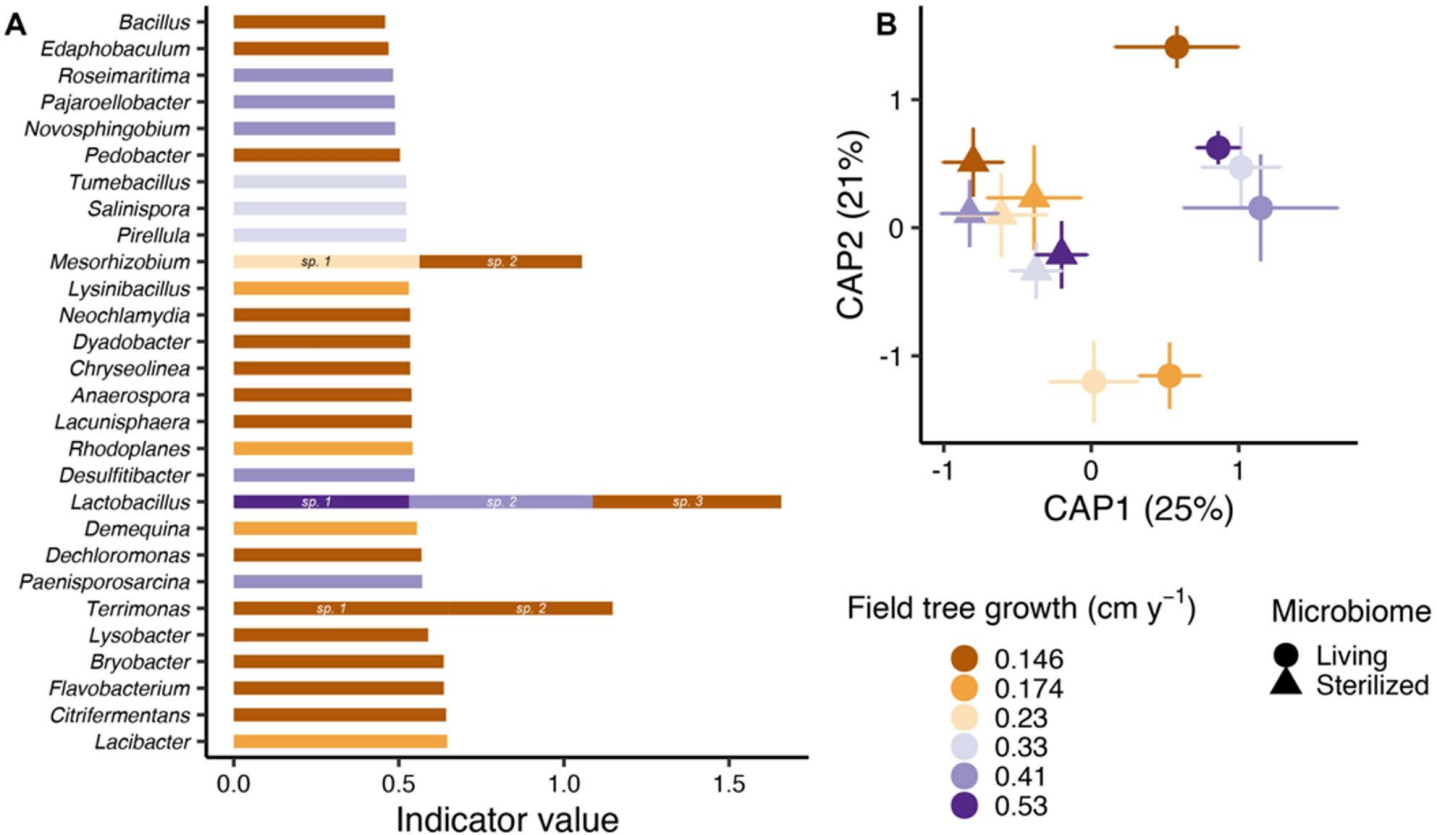
Bacterial community composition varies across sites and with field tree growth rate. Different bacterial species indicative of the sites from which inoculant was sourced with respect to field tree growth rate (**A**). Note that this analysis of indicator species was restricted to sites with living soil inoculant. Bacterial community composition visualized using constrained analysis of principal coordinates **B** showing significant differences in microbiome composition across sites based on variation in microbiome treatment (living versus sterilized) and field tree growth rates

Bacterial community composition, richness, and Shannon diversity were all resistant to elevated CO_2_ (Tables S2 and S3). However, unique ASVs drove variation in bacterial community composition across mesocosms according to the site from which inoculant was sourced (Fig. 3A, B). Bacterial community composition also differed between soil sterilization treatments (*P* = 0.03; Table S2). Communities in the sterilized treatment were homogeneous whereas communities in the living microbiome treatment varied across sites in relation to in situ field tree growth rates (Fig. 3A; *P* = 0.02). Together, these results demonstrate that distinct microbial communities of comparable diversity levels assembled in the mesocosms inoculated with different sources of living microbial inoculant. Interestingly, bacterial communities from the three fastest growing forests harbored similar communities with indicator species such as *Lactobacillus*,* Desulfitibacter*, and *Paenisporosarcina*.

**Fig. 4:**
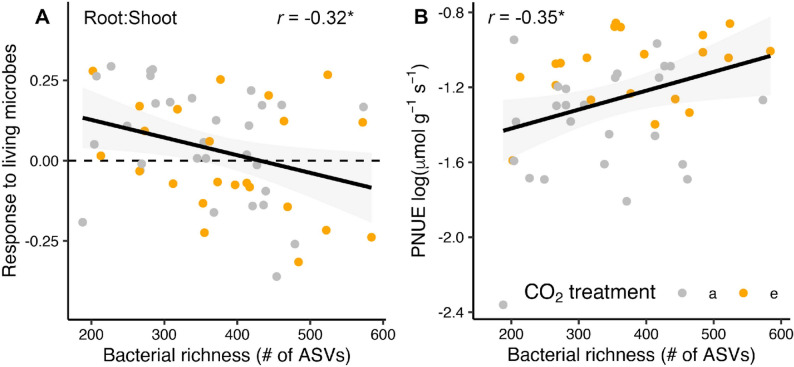
Linkages between tree seedling growth, physiology, and the bacterial community. Variation in root: shoot caused by differences in the microbiome, quantified as a response ratio between the living and sterilized conditions (see methods; **A**) under ambient (a) and elevated (e) CO_2_. Potential nitrogen use efficiency (PNUE) is positively correlated with bacterial richness (**B**). Lines represent linear correlations, shaded areas are 95% confidence intervals, r represents the Pearson correlation, and asterisks indicate *p*-values (* represents *P* < 0.05)

Variation in tree seedling growth and physiology across the different inoculation sources was correlated with soil bacterial diversity. Specifically, seedling root: shoot ratio response to living microbial inoculation was negatively correlated to bacterial richness in the living soil treatment (Fig. 4A). In other words, soil microbiomes with low bacterial diversity increased the response of root: shoot ratio to living microbes. In contrast, plant PNUE was positively correlated to bacterial diversity (Fig. 4B). When considered together, diversity of bacterial communities was positively linked to PNUE and negatively correlated with investment into root system biomass.

Saprotrophic fungal community composition was also correlated with seedling root growth and root: shoot. Note that we did not test for changes in saprotrophic fungal communities in response to the treatments (see Methods for more details). The most dominant saprotrophic fungi included *Cephalotrichum* (24% of sequences), *Zopfiella* (21%), *Arthrobotrys* (12%), *Lophotrichus* (11%), *Penicillium* (9%), and *Mortierella* (3%) (Supplementary Fig. 7). Specifically, saprotrophic fungal composition was correlated with root productivity responses to elevated versus ambient CO_2_ (Supplementary Fig. 8A), and it was positively correlated with root: shoot response to living versus sterilized soil inoculation (Supplementary Fig. 8B). In addition to bacterial species richness, variation in saprotrophic fungal composition further explains observed differences in plant productivity in mesocosms with different soil inoculant sources and atmospheric CO_2_ conditions.

### Soil inorganic nitrogen cycling shifts with microbial inoculation and eCO2 and is correlated with reduced seedling productivity

**Fig. 5:**
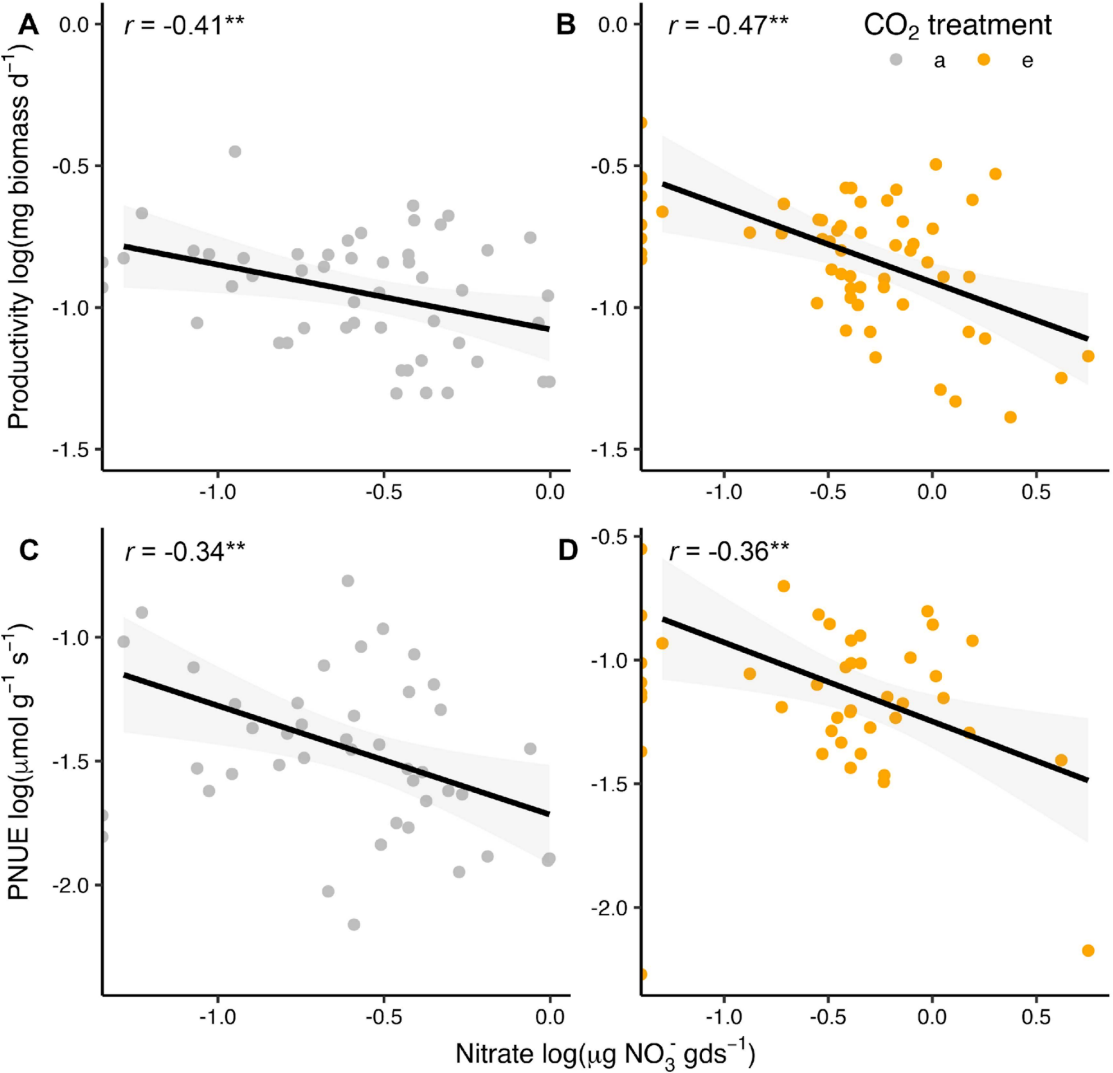
Links between soil nitrate concentrations and plant growth and physiology. Negative correlation between productivity and nitrate concentrations under ambient (**A**; referred to as ‘a’ in the legend) and elevated CO_2_ (**B**; referred to as ‘e’ in the legend), and its link to PNUE under ambient (**C**) and elevated CO_2_ (**D**). Lines represent linear correlations, shaded areas are 95% confidence intervals, r represents the Pearson correlation, and asterisks indicate *p*-values (** represents *P* < 0.001)

A central mechanism by which bacterial and saprotrophic fungal communities could affect seedling growth is mediation of soil inorganic N cycling. Total inorganic N availability was not significantly affected by eCO_2_ (*P* = 0.6; Supplementary Table 4). Nitrate alone was 441% higher under elevated versus ambient CO_2_ (*P* < 0.0001), but the magnitude of the eCO_2_-induced increase in nitrate varied by the living soil inoculant source (*P* < 0.0001). Ammonium availability also differed across the living soil inoculant sources, and this effect interacted with eCO_2_ (*P* = 0.01), with ammonium concentrations generally lower under elevated compared to ambient CO_2_. Ammonium was also a larger inorganic N pool than nitrate in the living soil inoculant treatments, with an average 4.2 µg gds^−1^ compared to 0.6 µg nitrate gds^−1^. While both pools of inorganic N varied with the soil inoculant source, nitrate was more responsive to eCO_2_ than ammonium. Nitrate, not ammonium, concentrations were also negatively correlated with seedling productivity (Fig. 5A, B) and PNUE (Fig. 5C, D), and this correlation was observed within, but not between the CO_2_, treatments due to elevated productivity and nitrate under eCO_2_. While there was no correlation to inorganic N cycling, soil pH was also significantly reduced by eCO_2_ (*P* < 0.001) and was comparable across mesocosms with different sources of soil inoculant (*P* = 0.08). This demonstrates that the stimulation of seedling productivity under eCO_2_ is negatively linked to increasing nitrate availability and that this is independent of soil pH.

## Discussion

In this study, we tested whether tree seedling optimization of physiological processes and growth were shaped by differences in the soil microbiome under changing atmospheric CO_2_ conditions. In the absence of a living microbiome, seedling growth was not only impaired but also significantly less responsive to eCO_2_ (Fig. 1A). In contrast, seedlings were always more productive under elevated versus ambient CO_2_ when inoculated with living soil; however, the magnitude of growth stimulation by eCO_2_ differed with the source of living soil inoculant (Fig. 1C). In fact, the effect of inoculating different soil microbiomes (i.e., different living soil inoculant sources) on plant productivity was even larger than a doubling of atmospheric CO_2_. Plant physiological responses to eCO_2,_ including carbon assimilation, PNUE, and biomass allocation below versus above-ground were also correlated with variation in soil bacterial and saprotrophic fungal communities. Our results demonstrate that plants can optimize growth under eCO_2_ by improving PNUE, and that PNUE is positively correlated with bacterial richness. While we cannot disentangle causality in our experiment, our results demonstrate that soils with more diverse bacterial communities are linked to greater plant optimization under eCO_2_, which may be a key mechanism to overcoming progressive N limitations induced by eCO_2_ in the future.

### Seedling productivity and responses to eCO_2_ were impacted by biological differences of the living microbial inoculant sources

Our full-factorial experimental design included sterilized soil treatments for each source of inoculant and CO_2_ level, which allowed us to compare and isolate soil biological versus physicochemical impacts on plant growth and responses to eCO_2_. Earlier research has demonstrated that variation in soil health is positively linked to plant productivity [42], and that this can be driven by microbial diversity, such as symbiotic rhizobia species richness [43] or by the abundances of particular taxa within the Firmicutes and Actinobacteria [44], with soil physical and chemical differences also playing important roles [45]. Independent contributions to plant growth by soil biological versus physicochemical attributes has been notoriously difficult to disentangle in earlier eCO_2_ experiments, but we were able to specifically isolate biological effects due to our study design. Both plant productivity and root: shoot ratio were homogenous across the mesocosms with sterilized soil inoculant compared to mesocosms with living soil inoculant. Bacterial community composition in the sterilized soil inoculant mesocosms was also homogenous and distinct from those in the living soil inoculant treatments. We can therefore conclude that there was little-to-no impact of soil physicochemical differences of the different field sourced soils on plant growth, which was expected because we deliberately used a small quantity of field soil for the inoculations to avoid this issue, but there were large differences in plant growth due to biological variation, supporting our first hypothesis.

Our second hypothesis was that seedling productivity would be correlated with the in situ field tree growth rates from the microbial inoculant source sites, but we found no evidence for this. In fact, seedling productivity was negatively correlated with field tree growth from the slowest to penultimate productivity site until spiking again at the fastest growing site. Thus, living soil inoculants sourced from both the slowest and fastest growing forests most strongly boosted seedling productivity. Interestingly, seedling productivity was negatively correlated with the N deposition rate at the field sites, but this effect was only marginally significant (*r* = -53, *p* = 0.08; Supplementary Fig. 9). This suggests that microbial adaptation (i.e., changes in community composition or physiology) to higher N deposition levels reduces plant growth promoting benefits. Previous studies have demonstrated that N deposition can reduce microbial biomass [46], suppress fungal diversity [47] and alter bacterial communities to select for faster growing taxa with reduced capacities for N relative to carbon uptake [48], all of which may reduce benefits conferred to growing plants. Thus, the field source site N deposition level, not tree growth rates, were better indicators of the effects of soil inoculation on plant growth in our controlled experiment. While earlier work has demonstrated that soil inoculation can drive specific plant community establishment [25] and growth rates [26], we were unable to recapitulate field observations of tree growth in our mesocosm experiment, rejecting our second hypothesis.

We, however, need to acknowledge that in contrast to the seedlings used here, trees in the field sites are established, adult individuals. Thus, long-term co-adjustment between trees and soil microbiomes occurred in the field and may explain why soil fungal communities were correlated with tree growth in earlier, observational studies [20], but not in our comparably short, seedling-focused experiment. Earlier investigations were also observational and could not evaluate causal effects of fungi on adult tree growth. Furthermore, several weeks passed between field soil collection and inoculation (though soil was kept cool at 4 °C), and microbial communities were transferred into novel physicochemical environments in the pots, both of which may have affected the biological viability of select members of the field soil microbial community. Even though we could not recapitulate observations from the field, our results provide strong evidence that variation in soil microbiomes cause distinct variation in plant growth rates, but we could not predict experimental effects on tree growth based on in situ field tree growth rates in this study system.

We identified bacterial ASVs significantly indicative of different soil inoculation source sites, and these taxa may be drivers of variation in plant growth rates in our mesocosm study system. For example, a *Lactobacillus* ASV was an indicator of plants growing with inoculant sourced from the fastest growing forest site, and taxa from this genus can promote plant growth and inhibit plant pathogens [49]. By proliferating in fast growing mesocosms, *Lactobacillus* may help to control opportunistic pathogens responding to root exudation in the rhizosphere while simultaneously having positive direct effects on plant growth (see review [50]). Many ASVs were indicators of plants growing with inoculant sourced from the slowest growing forest, which as described above, caused the fastest seedling growth in our mesocosm experiment. These included taxa with well-known positive effects on plant growth, including distinct *Lactobacillus* [49], *Bacillus* [51], *Flavobacterium* [52], and *Lysobacter* [53] ASVs. Groups like *Bacillus* include free-living N fixers [4], which obtain substantial energy from plant exudates. While these bacteria would hold onto any fixed N when alive, it would be released upon bacterial cell senescence and death, becoming subsequently available for plant uptake [54]. Saprotrophic fungal communities were also linked to variation in root growth responses to eCO_2_. By stimulating decomposition, saprotrophic fungi have been shown to influence patterns of tree seedling growth [55], and in this capacity, they can shape how much trees invest into resource capture via root growth. Some saprotrophic fungi may also be acting as endophytes [56], such as *Mortierella*, which were common in our study whereby they can directly shape root growth by increasing resource capture or inducing hormone production [57]. While we cannot identify whether specific bacterial or fungal taxa drove specific plant growth effects in our study because these were diverse communities with hundreds of species, accumulation of beneficial microbes at higher richness levels may explain why bacterial richness was positively correlated with PNUE and in turn overall plant productivity.

### Seedling growth increased under eCO_2_ and this response was driven by higher PNUE and microbiome variation

Elevated concentrations of atmospheric CO_2_ are widely expected to stimulate plant productivity [14, 58]. Consistent with many studies, we found enhanced plant productivity under eCO_2_ [59], altered root: shoot [60], greater C assimilation [61], and higher PNUE [16]. However, we demonstrate that the magnitude of these plant responses to eCO_2_ are directly affected by the soil inoculant source. We specifically observed higher PNUE as a driver of positive plant productivity responses to eCO_2_, and PNUE was positively correlated with bacterial taxonomic richness. This is consistent with other experimental work showing that bacterial taxonomic richness is positively linked to plant growth [62] and enhances decomposition and plant N uptake [63]. Stomatal conductance was also reduced under elevated versus ambient CO_2_ (Supplementary Table 1), consistent with prior reports in C_3_ species [6, 19]. However, the lack of a corresponding increase in WUE in our system suggests that enhanced carbon assimilation was not solely driven by stomatal regulation and may reflect microbial-mediated effects on photosynthetic capacity or internal N allocation [8, 9]. This may be stimulated under higher bacterial richness levels which can promote decomposition [64] and in turn nutrient availability. Bacterial richness was also negatively correlated with seedling root: shoot response to living versus sterilized soil inoculation, such that there were lower investments into root systems when bacterial diversity in the soil was high. Our results suggest that higher bacterial richness may promote plant growth responses to eCO_2_ by improving nutrient uptake and promoting the efficiency of growth limiting N use.

### Soil nitrate concentrations were negatively linked to plant growth responses to eCO_2_

The progressive N limitation hypothesis suggests that N availability will constrain plant growth responses to eCO_2_ overtime. While our short-term study was not designed to examine this phenomenon, by creating a relatively low-N soil environment where inorganic N levels were comparably low as observed in other *P. sylvestris* forests [65, 66], we could assess microbiome contributions to plant growth under typical N limiting conditions. Notably, we did not measure other forms of bioavailable N, such as amino acids. In our study system, eCO_2_ reduced plant N content by 19% (Supplementary Table 1). The tree species we studied, *P. sylvestris*, forms ectomycorrhizal symbioses [23]. Since some ectomycorrhizal fungi can mine soil organic matter for N [67], trees have been projected to potentially overcome progressive N limitations if they associate with ectomycorrhizal fungi [68]. While we were not able to link variation in seedling responses to eCO_2_ to ectomycorrhizal communities in our study (see Methods), we were able to explore how variation within the soil bacterial and saprotrophic fungal communities of *P. sylvestris* affect early tree life cycle feedback to inorganic N cycling. Our main finding shows that nitrate concentrations increased under eCO_2_ and that this was negatively correlated with plant productivity and PNUE.

Nitrate may have accumulated due to enhanced production by nitrifiers and/or reduced uptake by plants and microbes, including denitrifying bacteria There was no effect of eCO_2_ on net N mineralization (including net nitrification) nor the relative abundance of nitrifying bacterial ASVs (*P* >0.05), which suggests that nitrate did not accumulate because of enhanced production. Since net nitrification was measured in a separate laboratory incubation in the absence of plants (see methods), we cannot exclude the potential for reduced plant nitrate uptake during the experiment, and these findings may look different if we measured gross N mineralization. *P. sylvestris* more efficiently uses ammonium compared to nitrate [69]. In fact, when supplied exclusively with nitrate, *P. sylvestris* grows poorly and becomes chlorotic [70] due to over-accumulation of calcium, magnesium, and other cations [71] as well as iron chlorosis [70]. This could explain why nitrate availability was negatively correlated to PNUE in our study system. This finding suggests that nitrate accumulation under eCO_2_ may not only reflect an imbalance in soil nitrogen cycling but could also act as a physiological stressor. Whether this may occur under more realistic field conditions is an open question. Interestingly, a related study reported increased soil nitrate (and ammonium) levels under eCO_2_ in relation to enhanced N mineralization [1], but it further demonstrated that these responses were tree species specific, and *P. sylvestris* was not studied. This phenomenon may be due to the specific plant species we studied in addition to the watering regime of our experiment where soil was kept consistently moist but never saturated to maintain aerobic conditions. This could reduce denitrification [72], the main microbial process which removes nitrate from forest soil [73]. Thus, whether our results would occur in the field very likely depend on forest type, soil moisture availability, and its effects on denitrification.

### Study limitations

The major limitation of this study was our inability to test the link between EMF community composition and seedling productivity under eCO_2_. EMF community composition has been linked to variation in tree seedling growth in several studies (see review by [74]), and it was our original goal to link the entire soil microbiome, including EMF, to seedling growth and responses to eCO_2_. While we could only focus on bacterial and saprotrophic fungal communities in this study, it is possible that these groups may be responding to variation in plant growth due to changes in root exudation rates [75] or even in response to the functioning of EMF [76] versus contributing to tree growth via functions like nutrient cycling. However, saprotrophic fungi and bacteria are key mediators of decomposition [77] and nitrogen cycling [78], and they have been shown to play important roles in shaping inorganic N cycling, and in turn, progressive N limitations under eCO_2_ [79]. These mechanistic details cannot be teased apart in our study system, and because we also did not measure physiological rates in plants growing in the sterilized soil inoculant treatment, we cannot compare how functions, such as PNUE, differed in response to variation in living versus sterilized soil conditions. A deeper understanding of the microbial mechanisms would also require a more detailed characterization of most major soil nutrients versus our focus on nitrogen. While these limitations make it more challenging to identify causal mechanisms in our study system, they do not prevent us from concluding that variation in soil microbial communities were key drivers of seedling growth and responses to eCO_2_.

## Conclusions

Atmospheric CO_2_ levels are projected to increase dramatically into the future, and this is widely expected to promote plant productivity, provided sufficient supply of other nutrients. Here we provide strong evidence that microbiome community differences cause variation in *P. sylvestris* physiology, growth, and resource allocation above- and belowground. The impact of inoculating different microbial community types on plant productivity was more than two-times greater than a doubling of atmospheric CO_2_ concentrations, and the impact of microbes on plant growth was tightly linked to plant N use efficiency and energy allocation below- versus aboveground. Our results suggest that diverse microbiomes can optimize plant resource use, which may be especially important to sustain plant growth under eCO_2_. This microbial “buffering” may become increasingly relevant as long-term experiments indicate a risk of progressive N limitation under elevated CO_2_.

## Supplementary Information

Below is the link to the electronic supplementary material.

Supplementary Material 1

## Data Availability

Raw sequences are available in the NCBI SRA using accession numbers PRJNA1277171(16 S) and PRJNA1277183 (ITS). Analysis scripts and processed data are available on GitLab: https://gitlab.com/fungalecology/spmr.
