# Supplementary material for: Soil microbial community differences drive variation in Pinus sylvestris physiology, productivity, and responses to elevated CO2
Source: Environ Microbiome. 2025 Dec 4;21:3. doi: 10.1186/s40793-025-00828-w (PMC12797762; doi:10.1186/s40793-025-00828-w)
Supplement: Supplementary file 1 — Supplementary Material 1 [file 40793_2025_828_MOESM1_ESM.docx]

**Supplementary Materials**

**Soil microbial community differences drive variation in *Pinus sylvestris* physiology, productivity, and responses to elevated CO_2_**

Mark A. Anthony^1, 2, *, §^, Nora Röckel^2, *^, Alexandra Traistaru^2^, Aswin Krishna^2^, Henning Meesenburg^3^, Markus Wagner^3^, Frank Jacob^4^, Arthur Gessler^2, 5^, Peter Waldner^5^, Marcus Schaub^5^, Marco Ferretti^5^, Andreas Schmitz^6, 7^, Pim van den Bulk^8^, Arjan Hensen^8^, Stefan F. Hupperts^9^, Lalasia Bialic-Murphy^2^, Colin Averill^2, 10^

^1^Center for Microbiology and Environmental Systems Science, University of Vienna, Vienna, Austria.

^2^Department of Environmental Systems Sciences, ETH Zürich, Zürich, Switzerland

^3^Northwest German Forest Research Institute,Göttingen, Germany

^4^Sachsenforst State Forest, Pirna OT Graupa, Germany

^5^Swiss Federal Institute for Forest, Snow, and Landscape Research (WSL), Birmensdorf, Switzerland.

^6^ North Rhine Westphalia Office for Nature, Environment and Climate, Recklinghausen, Germany

^7^Thünen Institute of Forest Ecosystems, 16625 Eberswalde, Germany

^8^The Netherlands Organization for Applied Scientific Research at Petten, Petten, The Netherlands

^9^Department of Forest Ecology and Management, Swedish University of Agricultural Sciences (SLU), Umeå, Sweden

^10^Funga Public Benefit Corporation, Austin TX, USA

^*^M.A.A. and N.R. share co-first authorship

^§^Corresponding author e-mail: [mark.anthony@univie.ac.at](mailto:mark.anthony@univie.ac.at)

**Supplementary Table 1**. Summary statistics of plant growth responses to the treatments. Summaries of ANOVA with type III sums of squares are shown.

| **Aboveground productivity** | Sums of Sq | DF | F-value | P-value |
| --- | --- | --- | --- | --- |
| Inoculant source | 0.38159 | 5 | 40.3109 | 2.20E-16 |
| Sterilization | 0.6576 | 1 | 347.3368 | 2.20E-16 |
| CO2 | 0.10682 | 1 | 56.4239 | 3.39E-13 |
| Inoculant source x Sterilization | 0.15728 | 5 | 16.6146 | 5.12E-15 |
| Inoculant source x CO2 | 0.00996 | 5 | 1.0519 | 0.38661 |
| Sterilization x CO2 | 0.0579 | 1 | 30.5821 | 5.55E-08 |
| Inoculant source x CO2 x sterilization | 0.02024 | 5 | 2.1382 | 0.05997 |
| Residuals | 0.81789 | 432 |  |  |
|  |  |  |  |  |
| **Belowground productivity** |  |  |  |  |
| Inoculant source | 0.32431 | 5 | 26.5043 | 2.20E-16 |
| Sterilization | 0.82532 | 1 | 337.2526 | 2.20E-16 |
| CO2 | 0.03264 | 1 | 13.3365 | 0.0002922 |
| Inoculant source x Sterilization | 0.1942 | 5 | 15.8709 | 2.32E-14 |
| Inoculant source x CO2 | 0.00639 | 5 | 0.5225 | 0.7592943 |
| Sterilization x CO2 | 0.03034 | 1 | 12.3966 | 0.0004759 |
| Inoculant source x CO2 x sterilization | 0.00482 | 5 | 0.3937 | 0.8531596 |
| Residuals | 1.05719 | 432 |  |  |
|  |  |  |  |  |
| **Root:shoot ratio** |  |  |  |  |
| Inoculant source | 1.914 | 5 | 2.892 | 0.013968 |
| Sterilization | 2.262 | 1 | 17.0896 | 4.29E-05 |
| CO2 | 0.935 | 1 | 7.0659 | 0.008148 |
| Inoculant source x Sterilization | 6.358 | 5 | 9.6072 | 1.04E-08 |
| Inoculant source x CO2 | 1.038 | 5 | 1.5686 | 0.167633 |
| Sterilization x CO2 | 0.041 | 1 | 0.3084 | 0.578963 |
| Inoculant source x CO2 x sterilization | 0.148 | 5 | 0.2244 | 0.951923 |
| Residuals | 57.177 | 432 |  |  |
|  |  |  |  |  |
| **Assimilation rate** |  |  |  |  |
| Inoculant source | 471.88 | 5 | 14.4692 | 5.97E-12 |
| CO2 | 11.5 | 1 | 1.7628 | 0.18592 |
| Inoculant source x CO2 | 64.83 | 5 | 1.9878 | 0.08238 |
| Residuals | 1200.15 | 184 |  |  |
|  |  |  |  |  |
| **Water use efficiency** |  |  |  |  |
| Inoculant source | 0.679 | 5 | 1.4449 | 0.210414 |
| CO2 | 0.153 | 1 | 1.6312 | 0.203214 |
| Inoculant source x CO2 | 1.93 | 5 | 4.1055 | 0.001498 |
| Residuals | 16.544 | 176 |  |  |
|  |  |  |  |  |
| **Potential nitrogen use efficiency** |  |  |  |  |
| Inoculant source | 6.308 | 5 | 2.6825 | 0.02325 |
| CO2 | 2.629 | 1 | 5.5912 | 0.01921 |
| Inoculant source x CO2 | 1.823 | 5 | 0.7752 | 0.56887 |
| Residuals | 78.066 | 166 |  |  |
| **Stomatal conductance** |  |  |  |  |
| Inoculant source | 0.325 | 5 | 16.82 | 1.127E-13 |
| CO2 | 0.004 | 1 | 0.94 | 0.3329 |
| Inoculant source x CO2 | 0.068 | 5 | 3.52 | 0.0046 |
| Residuals | 0.71 | 184 |  |  |
|  |  |  |  |  |
| **Leaf N** |  |  |  |  |
| Inoculant source | 8.032 | 5 | 7.477 | 2.08E-6 |
| CO2 | 1.130 | 1 | 5.260 | 0.023 |
| Inoculant source x CO2 | 0.377 | 5 | 0.3508 | 0.881 |
| Residuals | 39.315 | 183 |  |  |

**Supplementary Table 2.** Summary statistics of bacterial community composition responses to the treatments, including field tree growth rate from which the inoculant was sourced as a predictor variable. Summaries are of distance-based redundancy analysis with bacterial composition computed as Bray-Curtis dissimilarities.

|  | DF | Sums of Sq | F-value | P-value |
| --- | --- | --- | --- | --- |
| CO2 | 1 | 0.2554 | 1.0162 | 0.368 |
| Sterilization | 1 | 0.3557 | 1.4155 | 0.037 |
| Field tree growth | 1 | 0.2438 | 0.9703 | 0.469 |
| Sterilization x CO2 | 1 | 0.1981 | 0.7884 | 0.9 |
| Field tree growth x CO2 | 1 | 0.2051 | 0.816 | 0.856 |
| Sterilization x Field tree growth | 1 | 0.4002 | 1.5926 | 0.019 |
| Sterilization x Field tree growth x CO2 | 1 | 0.2224 | 0.8852 | 0.687 |
| Residual | 106 | 26.6354 |  |  |

**Supplementary Table 3**. Summary statistics of bacterial alpha diversity responses to the treatments. Summaries of ANOVA with type III sums of squares are shown. Note that there was also no effect of field tree growth rate when replacing inoculant source in the statistical models.

| **Richness** | Sums of Sq | DF | F-value | P-value |
| --- | --- | --- | --- | --- |
| (Intercept) | 32.283 | 1 | 1674.7858 | <2e-16 |
| CO2 | 0.011 | 1 | 0.5616 | 0.4553 |
| Sterilization | 0.027 | 1 | 1.3781 | 0.2431 |
| Inoculant source | 0 | 1 | 0.0018 | 0.9659 |
| Sterilization x CO2 | 0.037 | 1 | 1.9292 | 0.1678 |
| Inoculant source x CO2 | 0.002 | 1 | 0.0835 | 0.7731 |
| Sterilization x Inoculant source | 0.044 | 1 | 2.3016 | 0.1322 |
| Sterilization x Inoculant source x CO2 | 0.036 | 1 | 1.8495 | 0.1767 |
| Residual | 2.043 | 106 |  |  |
|  |  |  |  |  |
| **Shannon index** |  |  |  |  |
| (Intercept) | 142.013 | 1 | 1397.1454 | <2e-16 |
| CO2 | 0.054 | 1 | 0.5311 | 0.4677 |
| Sterilization | 0.131 | 1 | 1.2929 | 0.2581 |
| Inoculant source | 0.009 | 1 | 0.0843 | 0.7721 |
| Sterilization x CO2 | 0.134 | 1 | 1.3229 | 0.2527 |
| Inoculant source x CO2 | 0.011 | 1 | 0.1058 | 0.7457 |
| Sterilization x Inoculant source | 0.245 | 1 | 2.4136 | 0.1233 |
| Sterilization x Inoculant source x CO2 | 0.129 | 1 | 1.2727 | 0.2618 |
| Residual | 10.774 | 106 |  |  |

**Supplementary Table 4**. Summary statistics of inorganic N cycling responses to the treatments. Summaries of ANOVA with type III sums of squares are shown. Values were first base-10 log transformed. Note that field tree growth rate was not correlated with any of the nutrient pools nor with N mineralization rate.

| **Total inorganic N** | Sums of Sq | DF | F-value | P-value |
| --- | --- | --- | --- | --- |
| Inoculant source | 1.3717 | 5 | 2.2685 | 0.04937 |
| Sterilization | 0.4013 | 1 | 3.3187 | 0.07007 |
| CO2 | 0.0185 | 1 | 0.1533 | 0.6958 |
| Inoculant source x Sterilization | 0.6534 | 5 | 1.0806 | 0.37245 |
| Inoculant source x CO2 | 0.7681 | 5 | 1.2703 | 0.27841 |
| Sterilization x CO2 | 0.0559 | 1 | 0.4625 | 0.49727 |
| Inoculant source x Sterilization x CO2 | 0.6986 | 5 | 1.1553 | 0.33284 |
|  |  |  |  |  |
| **Nitrate** |  |  |  |  |
| Inoculant source | 0.1681 | 5 | 1.0732 | 0.376572 |
| Sterilization | 0.0258 | 1 | 0.824 | 0.365158 |
| CO2 | 1.1266 | 1 | 35.9636 | 9.80E-09 |
| Inoculant source x Sterilization | 0.0429 | 5 | 0.2738 | 0.927031 |
| Inoculant source x CO2 | 0.5451 | 5 | 3.4799 | 0.004927 |
| Sterilization x CO2 | 0.499 | 1 | 15.9285 | 9.36E-05 |
| Inoculant source x Sterilization x CO2 | 0.2694 | 5 | 1.7202 | 0.131734 |
|  |  |  |  |  |
| **Ammonium** |  |  |  |  |
| Inoculant source | 1.0545 | 5 | 3.0231 | 0.01193 |
| Sterilization | 0.1855 | 1 | 2.6593 | 0.1046 |
| CO2 | 0.1596 | 1 | 2.2884 | 0.132 |
| Inoculant source x Sterilization | 0.5548 | 5 | 1.5905 | 0.1646 |
| Inoculant source x CO2 | 1.0553 | 5 | 3.0253 | 0.01188 |
| Sterilization x CO2 | 0.0364 | 1 | 0.5213 | 0.47118 |
| Inoculant source x Sterilization x CO2 | 0.622 | 5 | 1.783 | 0.11814 |
|  |  |  |  |  |
| **N mineralization^+^** |  |  |  |  |
| Inoculant source | 954.2 | 5 | 5.2912 | 0.0001614 |
| Sterilization | 40.4 | 1 | 1.1191 | 0.2917491 |
| CO2 | 21.3 | 1 | 0.5903 | 0.443446 |
| Inoculant source x Sterilization | 151 | 5 | 0.8372 | 0.5251826 |
| Inoculant source x CO2 | 757.6 | 5 | 4.2008 | 0.0013094 |
| Sterilization x CO2 | 118.2 | 1 | 3.2758 | 0.0722327 |
| Inoculant source x Sterilization x CO2 | 152.3 | 2 | 2.112 | 0.1244475 |

^+^Type II sums of squares computed, log values not used because some values were negative and the residuals of the raw data were normally distributed.

**Supplementary Fig. 1.** Community composition of EMF from the soil inoculation sites used for the study. Differences in EMF composition and site selection were determined in order to capture continuous variation in EMF community composition linked to variation in *in situ* forest productivity (sensu Anthony et al. 2022).


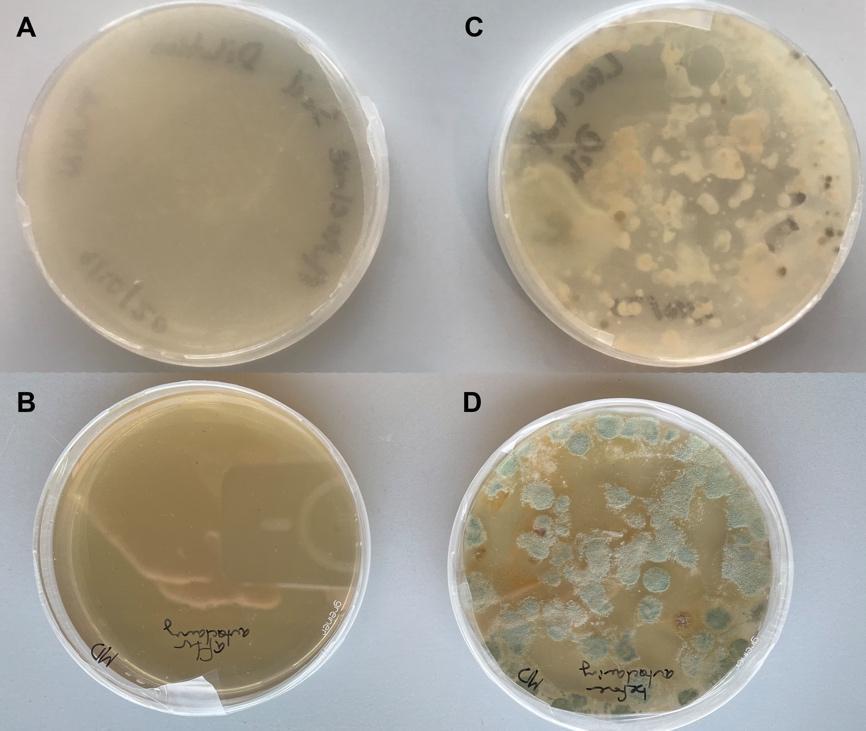


**Supplementary Fig. 2**. Visualization of petri-dish bioassay results testing the sterilization of soil following autoclave treatment. Sterilized soil (A, B) did not form any visible microbial colonies after two weeks whereas non-sterilized soil formed many colonies. Bioassays were performed using MMN media with low (2.5 mg glucose L-^1^; A-C) and high (10 mg glucose L-^1^; B-D) sugar contents.

**Supplementary Fig. 3. Variation in aboveground productivity in relation to the number of stems per pot in the autoclaved and living inoculation treatments.** There was no difference in aboveground productivity in relation to seedling density in the living inoculation treatment (*P* = 0.97). In the autoclaved treatment, aboveground productivity increased with seedling density (*P* = 0.002). There was no interaction between stem number and the other experimental factors (*P* > 0.05). There was no effect of stem number on root growth.

**Supplementary Fig. 4.** The relative abundance distribution of the top 100 fungal OTUs in the study system. Bars show the mean relative abundance in the entire sequencing dataset. OTUs assigned to fungi at the kingdom level but not at deeper taxonomic resolutions are indicated as “k__Fungi”. OTUs are annotated to the highest taxonomic rank possible. Note that black bars within a larger set of stacked bars indicate different OTUs. Most identified fungi in the dataset are saprotrophs, and none of the top 100 OTUs include EMF.


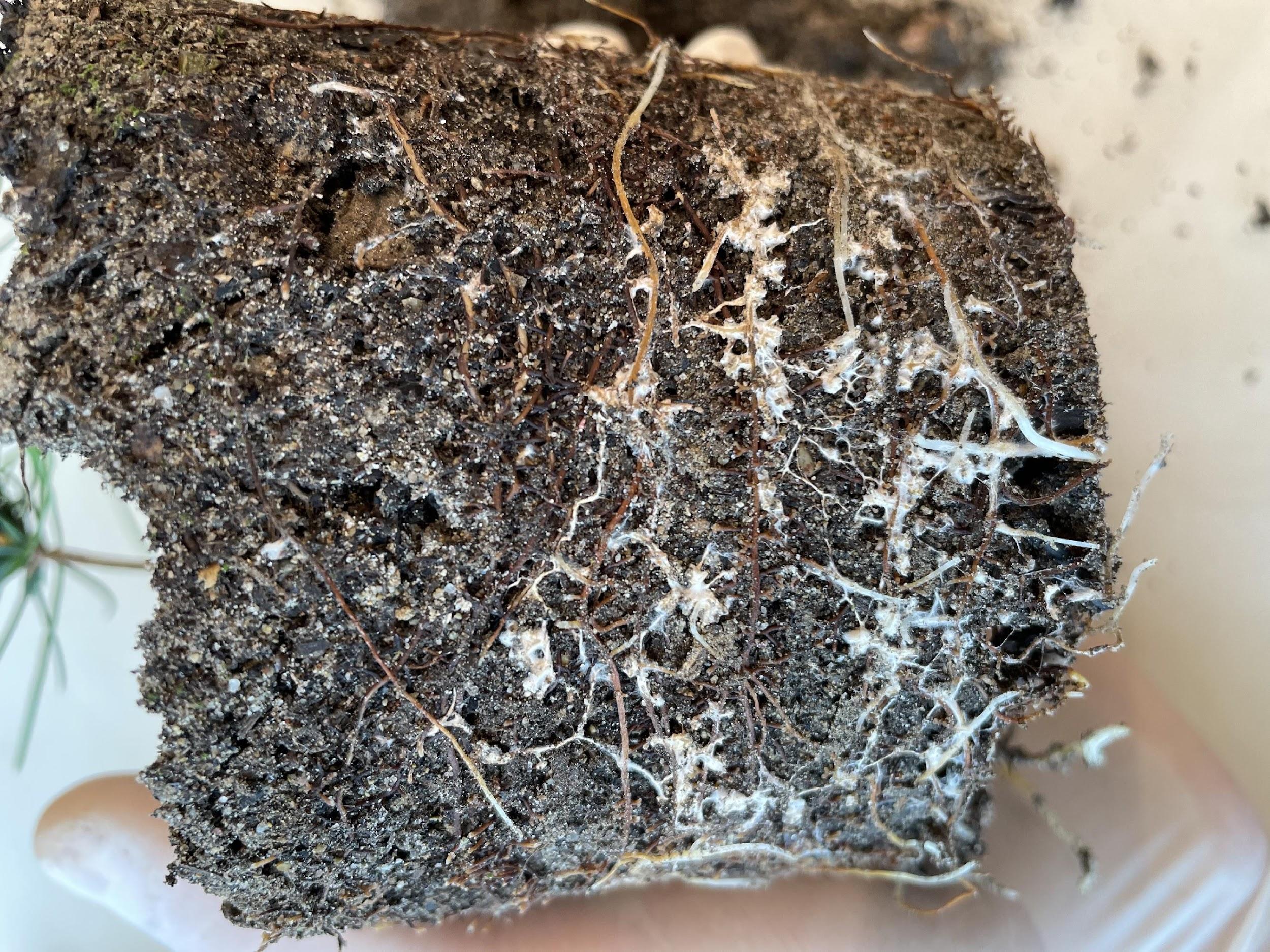


**Supplementary Fig. 5.** Pine root system heavily colonized by ectomycorrhizal fungi from the living inoculant. Despite visible mycorrhizal colonization, we detected very few, low relative abundance ectomycorrhizal fungal OTUs in the soil ITS metabarcoding dataset.


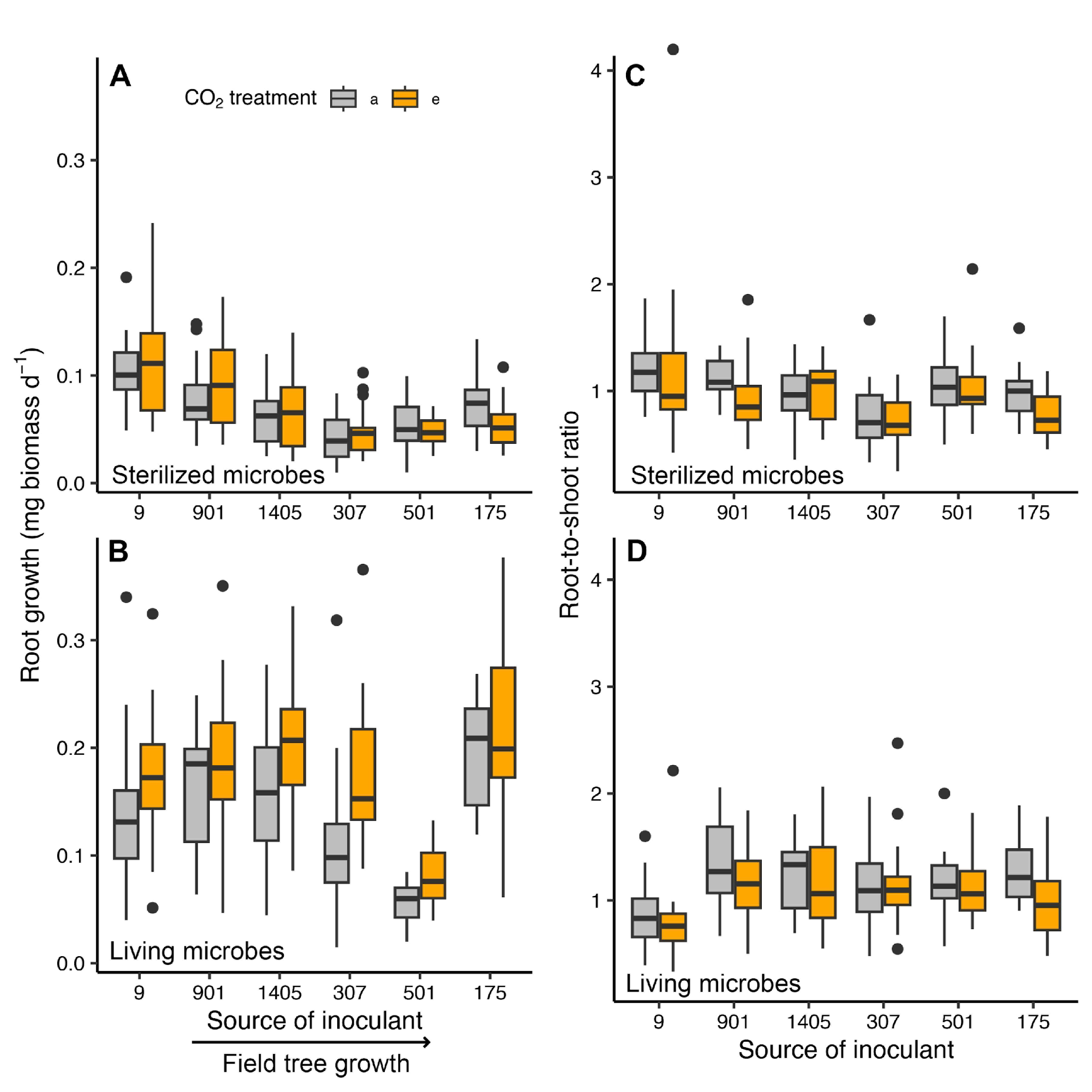


**Supplementary Fig. 6**. Root productivity and root:shoot ratio across the treatments and under ambient (a) and elevated (e) CO_2_. See Supplementary Table 1 for summary statistics.

**Supplementary Fig. 7.** Mean relative abundances of saprotrophic fungi. Bars show the mean relative abundance across all treatments with living soil inoculant. Note that this Fig. is only descriptive to provide an overview of the taxonomic composition of fungi in the dataset.


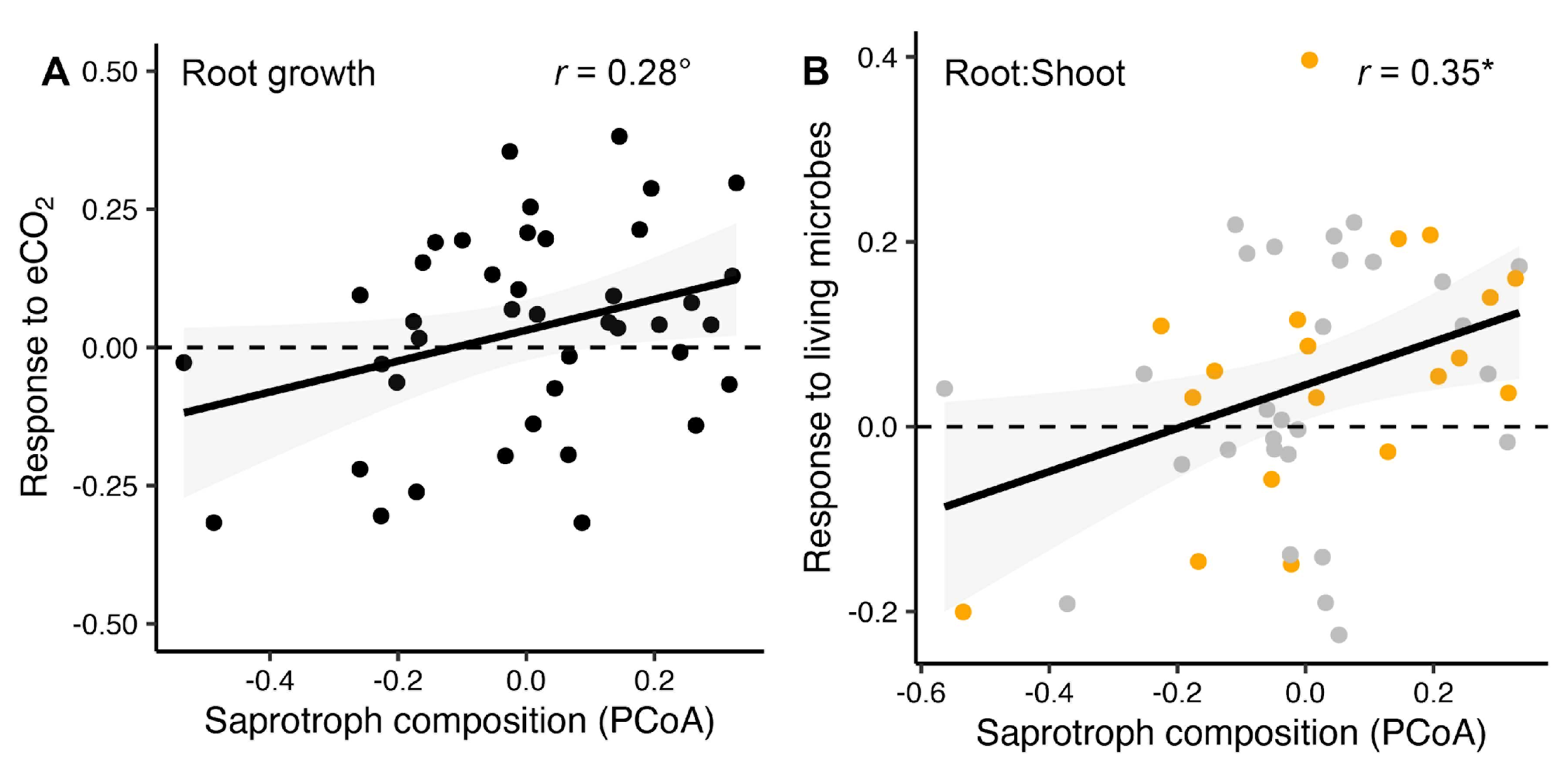


**Supplementary Fig. 8.** Linkages between tree seedling growth and the saprotrophic fungal community. Root productivity response to elevated CO2, quantified as a response ratio between the elevated and ambient conditions, is positively correlated with bacterial community composition as the second principal coordinates analysis axis (see methods; A). Correlations only show mesocosms with living microbial inoculant. Variation in root:shoot ratio caused by differences in the microbiome, quantified as a response ratio between the living and sterilized conditions, is positively correlated with saprotrophic fungal community composition (B). Lines represent linear correlations, shaded areas are 95% confidence intervals, r is the Pearson correlation, and asterisks indicate significance (o represents a marginally significant effect: P < 0.06; * represents P < 0.05).


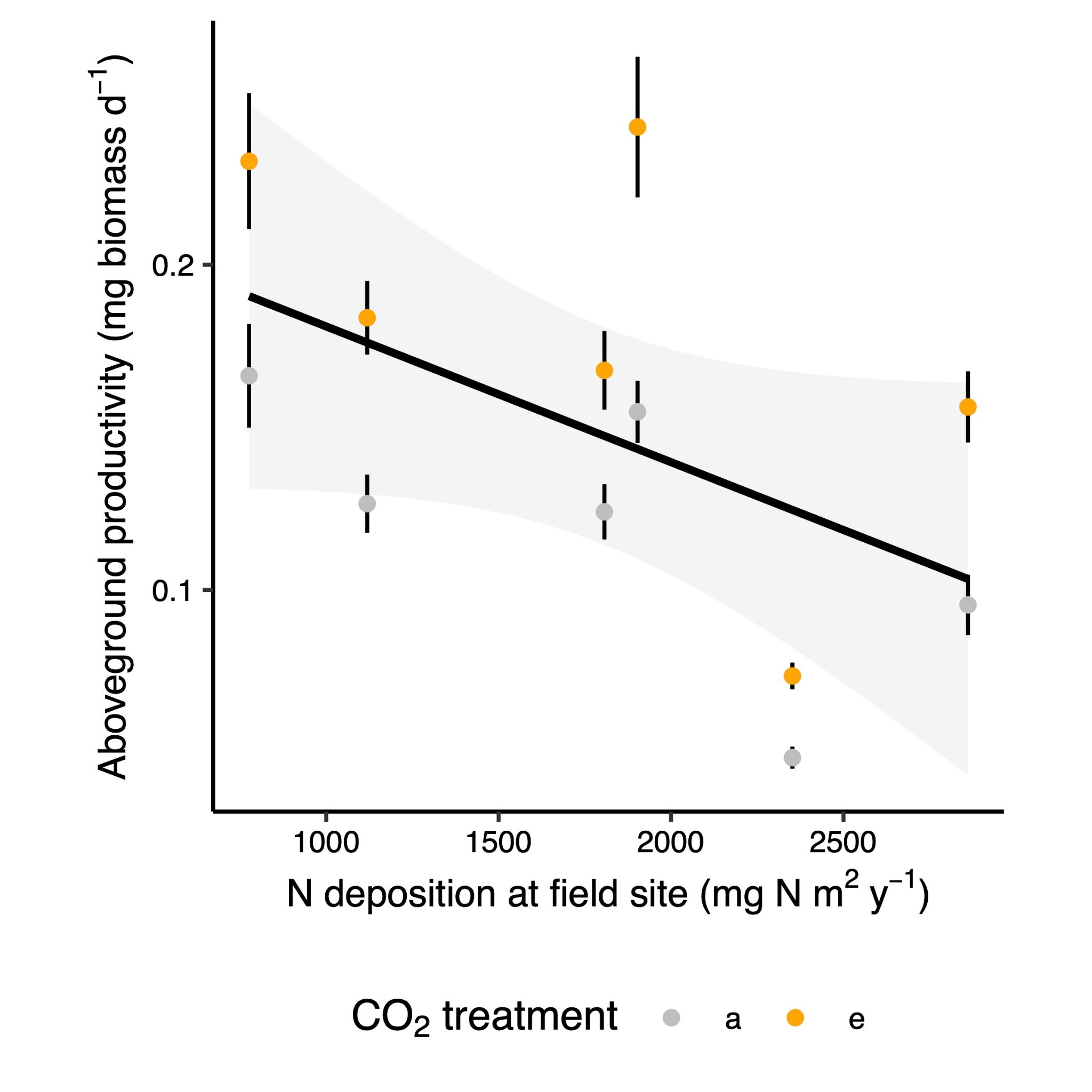


**Supplementary Fig. 9.** The correlation between aboveground plant productivity and N deposition rate at the field site (*r =* -0.53, *p* = 0.08). Note that only data form the living microbiome inoculation treatment was included. Points represent the mean and error bars are the standard error.
